# Recurrent vaginal evisceration of abdominal contents with subsequent resection of necrotic omentum in a 35-year-old woman

**DOI:** 10.1093/jscr/rjae127

**Published:** 2024-03-07

**Authors:** Crista E Horton, Ahmad Baghdadi, Jennine Putnick, Trevor Gravely

**Affiliations:** Broward Health Medical Center, Department of Surgery, 1600 S Andrews Ave, Fort Lauderdale, FL 33316, United States; University of Miami, Department of Surgery, 1611 NW 12^th^ Ave, East Tower 2169, Miami, FL 33136, United States; Broward Health Medical Center, Department of Surgery, 1600 S Andrews Ave, Fort Lauderdale, FL 33316, United States; Broward Health Medical Center, Department of Surgery, 1600 S Andrews Ave, Fort Lauderdale, FL 33316, United States

**Keywords:** vaginal evisceration, vaginal cuff dehiscence, recurrent vaginal evisceration, surgical repair vaginal evisceration

## Abstract

Vaginal evisceration is a rare surgical emergency in which intra-abdominal contents protrude through a dehisced vaginal cuff, which can lead to bowel ischemia and abdominal sepsis. This condition occurs due to vaginal cuff weakness secondary to prior surgeries or trauma. Recurrence after repair is rare and few cases have been documented. Here we present a young woman with multiple prior gynecologic surgeries who presented with eviscerated small bowel and omentum from her vagina five months following surgical treatment of a previous vaginal evisceration. Via a transabdominal surgical approach, general surgery and gynecology teams reduced the intra-abdominal contents, resected a pedicle of necrotic omentum, suture repaired the vaginal cuff, and placed a dehydrated placental allograft. This extremely rare case of recurrent vaginal evisceration demonstrates the importance of taking appropriate preventative surgical measures, maintaining a healthy level of suspicion for recurrence, knowing potential complications, and educating patients to prevent recurrent vaginal evisceration.

## Introduction

Vaginal evisceration is a rare condition and surgical emergency in which intra-abdominal contents protrude through the vaginal canal [1]. This condition occurs following full or partial dehiscence of the vaginal cuff [2]. Vaginal evisceration most commonly occurs as a post-hysterectomy complication or obstetric trauma and has an estimated incidence of 0.032%–1.2% after hysterectomy, trachelectomy, or upper vaginectomy [3]. Most patients present with bleeding, pain, pressure, or a protruding mass, and most patients present for medical care within 24 hours of symptom onset [4]. Complications associated with this condition include bowel ischemia and abdominal sepsis, as small bowel is the most common organ to eviscerate and become strangulated [5]. Although the predominant stressors that precede vaginal evisceration stratify according to menopausal status, with sudden increase in intra-abdominal pressure the most common cause in postmenopausal women and rough sexual intercourse the most common cause in premenopausal women, these causes do not account for many observed cases, as many women are asymptomatic with no identifiable precipitating event [2].

Despite the incomplete clinical picture accompanying many cases of vaginal evisceration, some risk factors have been identified. These risk factors include vaginal cuff weakness or atrophy secondary to increased age, obesity, prior vaginal surgeries, factors for poor wound healing (including malignancy, chronic steroid use, malnutrition, or tissue radiation) chronic Valsalva associated with coughing or constipation, vaginal cuff infection or hematoma, obstetric maneuvers, and vaginal instrumentation [2, 6].

Vaginal evisceration demands surgical intervention. Several surgical approaches for repair have been described, some involving combined open abdominal and transvaginal approaches. Others employ combined laparoscopic and transvaginal approaches, with or without omental flap, mesh, or sacro-colpopexy [7, 8]. In general, surgical repair is durable, as recurrence of vaginal evisceration after initial surgical repair is rarer than a single occurrence [9, 10]. This case describes an obese, premenopausal woman who experienced a recurrent vaginal evisceration five months after surgical repair with protrusion of small bowel and omentum. This recurrence was treated with an open transabominal approach. This unique case necessitated resection of necrotic omentum identified after restoration of eviscerated contents to the abdomen.

## Case report

**Figure 1:**
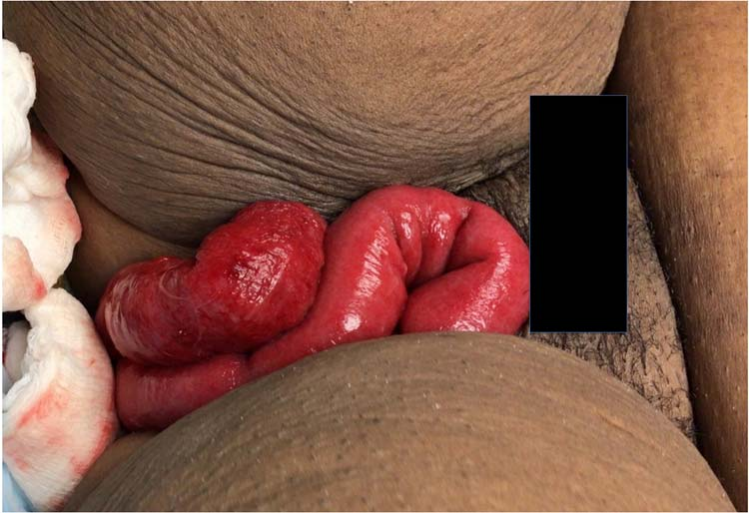
Evisceration of small bowel from vagina in a 35-year-old woman on initial presentation to the emergency room.

A 35-year-old woman presented to the emergency room with severe lower abdominal pain and evisceration of bowel contents from her vagina after urinating. She had a history of prior vaginal evisceration repaired surgically five months prior, hysterectomy, four caesarian sections, and Roux-en-Y gastric bypass. She reported experiencing persistent symptoms of urinary incontinence, dyspareunia, and abdominal pain during the past five months. One month prior to her second presentation to the emergency room for vaginal evisceration, she underwent two outpatient silver nitrate treatments to her vaginal cuff with her obstetrician/gynecologist (ob/gyn) due to vaginal bleeding and dyspareunia. In the emergency room, she had mild abdominal pain, with small bowel eviscerated from her vagina, as shown in Fig. 1. The bowel had appropriate peristalsis and demonstrated no evidence of perforation or strangulation. She remained hemodynamically stable with no leukocytosis or acidosis. She received intravenous fluids, antibiotics, and a multimodal pain regimen.

Prior to initial surgical consultation, her condition was thought to be rectal prolapse and was managed with attempts at reduction following application of sugar. Once evaluated by ob/gyn and surgery teams, however, her eviscerated bowel was covered in warm saline gauze for protection. The ob/gyn team planned for emergent operative intervention and consulted general surgery for co-management and possible bowel resection if indicated.

**Figure 2:**
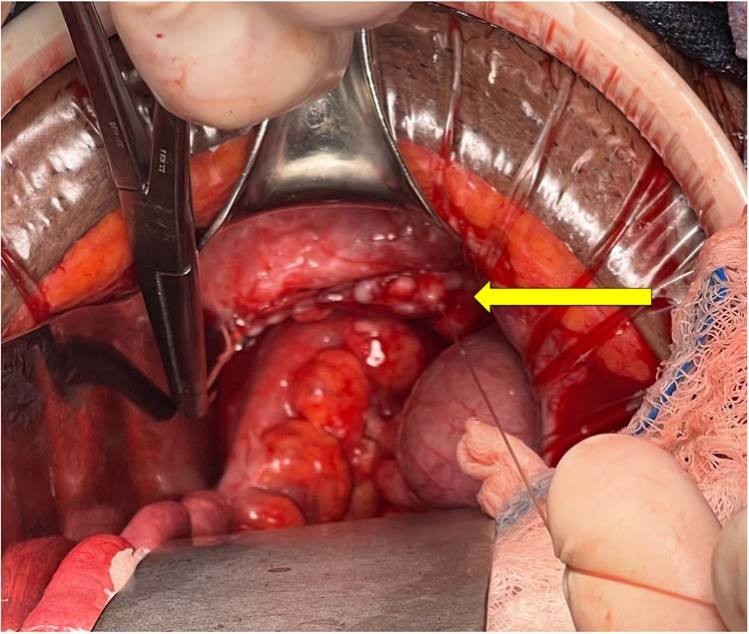
Intraoperative view of vaginal cuff repair after reduction of small bowel contents and resection of necrotic omentum. The vaginal cuff is indicated by the yellow arrow.

In the operating room, the abdominal cavity was opened via a Pfannenstiel incision, and the incarcerated small bowel and omentum were immediately reduced through the vagina. There was a long omental pedicle with small areas of ischemia at the edges, which were removed with electrocautery and sent for specimen. The rest of the omentum and small bowel appeared healthy. The ob/gyn surgeon decided that the vagina was already short and did not want to shorten it further, risking a fistula to the bladder, so the vaginal cuff was closed with synthetic, interrupted absorbable monofilament sutures primarily. Then, a dehydrated placental allograft wound covering was placed over the vaginal cuff, followed by an omental patch. The edges of the vagina appeared infected as seen in Fig. 2, so it was recommended that she allow time to heal with no heavy lifting, straining activities, or vaginal intercourse for 12 weeks. The patient did well postoperatively and was discharged on hospital Day four.

## Discussion

Vaginal evisceration is a rare, potentially life-threatening surgical emergency and must be promptly recognized and treated to prevent significant morbidity. While there has been some evaluation of optimal surgical approaches for this condition, there has been minimal investigation into treatment of patients with recurrence. In a similar case of recurrent vaginal evisceration following a gynecologic surgery, the surgeons conducted a laparoscopic sacro-colpopexy with placement of polypropylene mesh with success [9]. In contrast, for the case presented here, an open approach was utilized for timely and adequate exposure in preparation for potentially emergent resection of necrotic contents and simultaneous repair of the vaginal cuff. It is critical for surgical teams to prevent recurrence by appropriately closing the vaginal cuff during initial surgery, and to recognize vaginal evisceration as a potential high-risk complication. Clinicians should carefully determine a patient’s potential to experience vaginal evisceration based on patient history and physical exam. It is plausible that in this patient’s case, the use of silver nitrate after initial surgery may have contributed to poor wound healing and trauma to the vaginal cuff, leading to recurrent dehiscence. Additionally, it is crucial to understand that other pathologies such as rectocele, rectal prolapse, or cystocele may mimic this condition. Long-term follow-up and counseling may be necessary to reduce the risk of further recurrence and to improve the patient’s quality of life. Providers should also strive to educate their patients regarding temporary postsurgical sexual abstinence to allow adequate healing. This case report highlights a case in which a dangerous and rare occurrence of recurrent vaginal evisceration was successfully managed and provides a blueprint for managing such rare conditions.
